# Choanal stenosis post radiotherapy for nasopharyngeal carcinoma: about an endoscopic management

**DOI:** 10.11604/pamj.2019.34.111.18968

**Published:** 2019-10-25

**Authors:** Youssef Lakhdar, Sara Rochd, Mohamed Elbouderkaoui, Youssef Rochdi, Hassan Nouri, Abdelaziz Raji

**Affiliations:** 1 ENT-HNS Department, Mohammed VI University Hospital Center, Marrakech, Morocco

**Keywords:** Choanal stenosis, nasopharyngeal cancer, radiotherapy, endoscopic surgery

## Abstract

Choanal atresia is a rare complication of radiation for nasopharyngeal carcinoma, which has to be early detected. Its treatment is based on endoscopic endonasale surgery. We report a rare case of choanal stenosis observed in a 54-year-old patient, Ho presented 4 years after the end of radiotherapy for nasopharyngeal carcinoma, a progressive bilateral nasal obstruction, anosmia, and rhinorrhea without bleeding. The diagnostic of fibrous stenosis was confirmed by endonasal endoscopic examination coupled to CT scan of nasopharynx. The recanalization via endoscopic endonasal surgery with tube calibration gave a great functional result with the improvement of nasal symptoms. Even after 6 months of follow-up, there were no signs of restenosis.

## Introduction

Choanal atresia is a common congenital malformity in newborns; it is characterized by a blocked choanae, the opening between the nose and the nasopharynx. Acquired choanal stenosis is rarely seen and those complicating radiotherapy is even rarer in the literature. Through this observation we describe the clinical presentation, the diagnostic means and the therapeutic value of endoscopic recanalization surgery.

## Patient and observation

**Figure 1:**
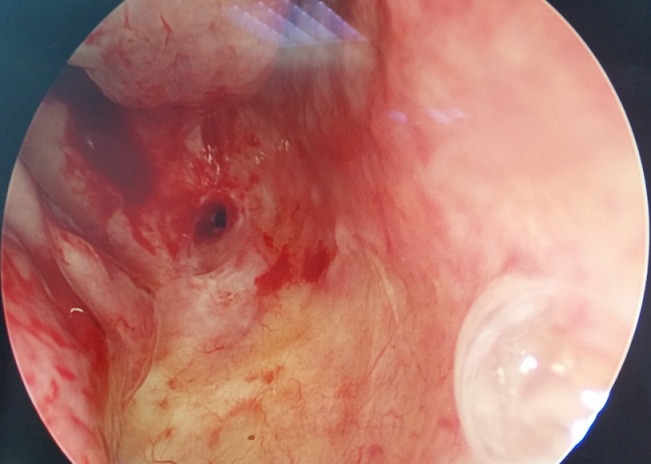
Endoscopic examination showing an obstructive stenosis of the choanae

**Figure 2:**
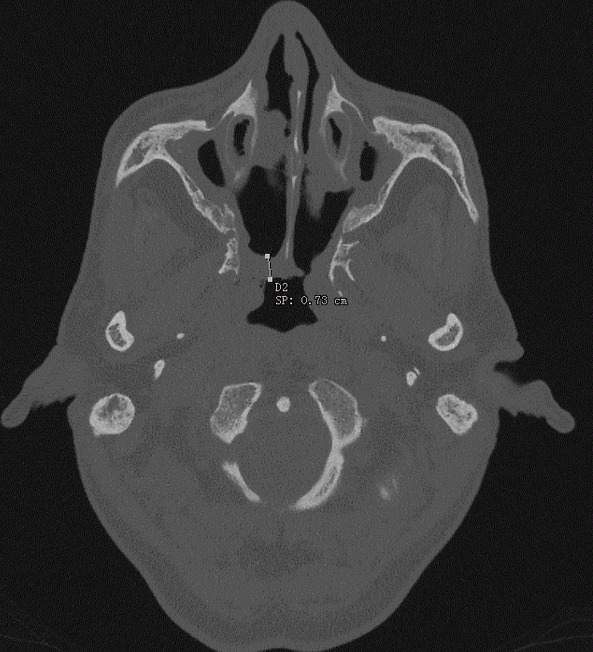
Facial CT scan in the axial section showing a stenosis of both choanae

A 54 years old Moroccan man presented to our otolaryngology (ENT) department with a 5 months history of worsening bilateral nasal obstruction, anosmia and rhinorrhea from both nostrils without epistaxis, associated with progressive bilateral hearing loss. His past medical history included undifferentiated carcinoma of the nasopharynx (UNCT) diagnosed on 2001, American Joint Committee for Cancer TNM staging of T1N0M0. He received a course of radiotherapy (65 Grays). The patient had no other significant past medical history. On examination, patient was breathing with mouth open. A cold spatula test showed absence of air flow on left nostril and minimal air flow on the right side. Rigid endoscopic nasal examination revealed a complete choanal stenosis in the left side and incomplete in the right side. There were no signs of tumor recurrence (Figure 1). Otoscopic examination showed signs of fibro-adhesive otitis and tonal audiometry showed mixed deafness. The computed tomography (CT) scan of the nose and paranasal sinuses was done, and showed a stenosis of the light of the two choanae by tissue density lesion in both choanae of 7.30mm (Figure 2). The patient underwent endoscopic surgery under general anesthesia, where the fibrous obstacle was resected and widening of the posterior choanal stenosis. A 5mm diameter tube was placed in both nasal cavities and kept for 3 weeks to ensure a good calibration and prevent the recurrence of the stenosis. The anatomopathological study did not show signs of tumor recurrence or specific inflammation. Deafness has been treated by bilateral hearing aids. The evolution was marked by the improvement of the clinical symptomatology with restitution of the nasal ventilation and the smell. An endoscopic control after 6 months of the surgical procedure did not find any sign of restenosis with a rather satisfactory choanal light.

## Discussion

Nasopharyngeal cancer is a public health problem in South-east Asia and in the Maghreb countries [1], its incidence varies between 30-80/100000/year in areas of high endemicity, including North Africa [2]. These nasopharyngeal cancers are treated mainly by radiotherapy, and their follow-up is based on clinical, endoscopic and radiological monitoring, which is often lacking in our context. This radiation therapy at curative doses can cause several sequels related to its local toxicity, choanal stenosis, although if it's rare, is part of it [3]. Only a few cases of choanal stenosis after radiotherapy have been reported in the literature, this may be explained by a lack of follow-up in these patients. A study carried out in 2014, studying the incidence of various late nasal-sinus post radiation complications, showed that 15% of patients developed choanal stenosis [4]. The clinical profile of our patient is similar to data reported in the literature: The average age found was 40 years [5-7] with a revealing symptomatology with most found signs were: nasal obstruction, anterior rhinorrhea and anosmia [5,6]. Rigid endoscopy allows early diagnosis at the clinical stage and CT allows confirming and quantifying the degree of stenosis [5-8]. In the literature this stenosis was bilateral complete or not in 84% of cases [4-8], and unilateral in 16% [8,9]. the treatment of choice for this complication is video-assisted endonasal surgical permeabilization with nasal cavity calibration, maintained for 2 to 6 weeks [5-8]. However surgery by micro endoscopic debridement without postoperative calibration can give satisfactory results without signs of restenosis [9,10]. In our case, the endoscopic surgery, with a calibration for 3 weeks, allowed us a functional improvement of the patient and a complete regression of the symptomatology, without signs of restenosis to endoscopic control at 6 months.

## Conclusion

Post radiation choanal stenosis is an exceptional complication. Its treatment is based on endonasal video surgery associated with a calibration. Endoscopic surveillance is mandatory, in addition to imaging, in irradiated nasopharyngeal cancers, to ensure proper monitoring and early detection of this complication.
